# Porous silver-coated pNIPAM-*co*-AAc hydrogel nanocapsules

**DOI:** 10.3762/bjnano.10.194

**Published:** 2019-10-04

**Authors:** William W Bryan, Riddhiman Medhi, Maria D Marquez, Supparesk Rittikulsittichai, Michael Tran, T Randall Lee

**Affiliations:** 1 Department of Chemistry and the Texas Center for Superconductivity, University of Houston, 4800 Calhoun Road, Houston, TX 77204-5003, United States

**Keywords:** hydrogels, nanocapsules, photothermal delivery, poly(NIPAM), porous silver shells

## Abstract

This paper describes the preparation and characterization of a new type of core–shell nanoparticle in which the structure consists of a hydrogel core encapsulated within a porous silver shell. The thermo-responsive hydrogel cores were prepared by surfactant-free emulsion polymerization of a selected mixture of *N*-isopropylacrylamide (NIPAM) and acrylic acid (AAc). The hydrogel cores were then encased within either a porous or complete silver shell for which the localized surface plasmon resonance (LSPR) extends from visible to near-infrared (NIR) wavelengths (i.e., λ_max_ varies from 550 to 1050 nm, depending on the porosity), allowing for reversible contraction and swelling of the hydrogel via photothermal heating of the surrounding silver shell. Given that NIR light can pass through tissue, and the silver shell is porous, this system can serve as a platform for the smart delivery of payloads stored within the hydrogel core. The morphology and composition of the composite nanoparticles were characterized by SEM, TEM, and FTIR, respectively. UV–vis spectroscopy was used to characterize the optical properties.

## Introduction

Interest in the preparation and utilization of uniquely structured nanocomposites continues to expand due to their potential use in electronics, optics, magnetism, medicine, catalysis, and energy applications [1–6]. The physical and chemical properties of these materials provide significant advantages over bulk materials in terms of reactive surface area, mobility, carrier capacity, bioavailability, and absorption/scattering across a broad range of wavelengths, even into the near-infrared (NIR) [7–10]. Nanostructured composites have been reported in a variety of shapes, sizes, and compositions [11–15]. Core–shell nanostructured composites have been the focus of recent work due to their structural simplicity and ability to introduce multifunctional properties into their structure [16–20]. One typical structure is that of an exogenous spherical capsule containing various core materials [21–24]. Recent studies involving spherical capsules have introduced a variety of materials into the core such as DNA, antibiotics, fluorescent dyes, and metal nanoparticles [25–30]. These types of particles show great promise for applications such as drug delivery, biosensing, chemical separation, nanoscale reactors, and catalysis [31–40].

While these examples represent a vast array of potential applications using nanocapsules, one particularly interesting application involves the use of NIR-responsive metal nanoparticles to create photothermally active biomaterials [41–43]. Recently, metal nanoshells, a class of optically active core–shell metal nanoparticles, have drawn interest, not only because of their ability to interact with light across a wide range of visible and NIR wavelengths, but also because of their enhanced extinction compared to molecular chromophores [44–45]. This type of particle generally consists of a single metal or an alloy shell with or without a dielectric silica core [46–48]. Fortunately, for biological applications, the optical properties of the nanoshells can be tuned by varying the particle composition, core size, and/or shell size to absorb/scatter wavelengths in the NIR region, which is largely transparent to human tissue [49–51].

Thermo-responsive hydrogel polymers have been extensively studied and have been utilized in various technological applications such as drug delivery, chemical separation, and catalysis [52–56]. These materials owe their technological importance to thermally induced structural changes [55]; for example, aqueous hydrogel solutions undergo volume transitions dependent upon their chemical or physical environments. This activity can be defined by the lower critical solution temperature (LCST); that is, the temperature at which the hydrogel polymers become hydrophilic and soluble in aqueous solutions, or conversely, hydrophobic and insoluble in aqueous solutions [57–59]. Interestingly, through chemical modification, the LCST can be adjusted, thus allowing thermally induced structural changes to occur over a wide range of temperatures rather than at one specific temperature. In particular, when poly-*N*-isopropylacrylamide (pNIPAM)-based hydrogels are co-polymerized with acrylic acid (pNIPAM-*co*-AAc) or acrylamide (pNIPAM-*co*-AAm), the LCST of the hydrogel copolymers can be tuned from ≈30–60 °C [60–61]. As such, hydrogel copolymers are capable of undergoing completely reversible swelling–collapsing volume transitions in response to changes in temperature [62–64]. Appropriate chemical modifications to the thermo-responsive hydrogel polymers allow researchers to tailor the LCST to biologically relevant temperatures.

To this end, researchers have made significant progress toward creating photothermally responsive hydrogel-based materials that respond to variations in temperature and/or pH [61,65–68]. For example, Zhao et al. created pH- and temperature-sensitive bioprobes by incorporating pNIPAM hydrogel cores with europium organic complexes [67]. In separate studies, Lee and co-workers utilized both biocompatible gold nanoshells, iron oxide nanoparticles, and gold nanorods with thermo-responsive hydrogel polymers to create a nanocomposite system in which the outer polymer shell can be thermally activated by absorption of light or by a magnetic field [41,61].

By combining the optical properties of nanoshells and the thermo-responsive activity of hydrogel polymers, the present study seeks to develop a nanoscale delivery system that can be targeted, optically activated, and subsequently release a payload (e.g., drugs). Notably, gold and silver nanoshells can be heated by application of NIR light and subsequently be used to transfer heat to an incorporated hydrogel polymer particle [69]. Upon heating, these hydrogel polymers shrink and release water and other materials encapsulated inside. Typically, gold, silver, and copper are the most commonly used materials for NIR activation, with silver being intermediate in both cost and oxidation resistance. Previous efforts have reported the combination of hydrogel and polymer nanocomposites with silver nanoparticles [70–73]. However, to the best of our knowledge, there have been no reports of NIR-active porous silver nanocapsules containing thermo-responsive hydrogel (or any kind of polymer) cores. The methods described herein illustrate the encapsulation of thermo-responsive pNIPAM-*co*-AAc hydrogel cores within porous silver nanoshells, and for the purpose of comparison, within a complete nonporous silver nanoshell. We adopt a simple surfactant-free emulsion polymerization (SFEP) technique to grow the initial hydrogel core templates [74–76]. Additionally, we use previously established seeded-growth methods to accomplish complete encapsulation of the hydrogel core within the porous and nonporous silver shells [77]. We envision that these types of particles will be technologically important for the future development of "smart" delivery and release vehicles for a variety of payloads.

## Results and Discussion

**Scheme 1:**
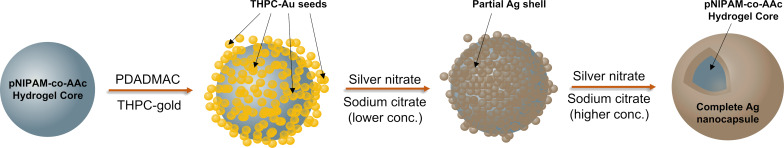
Strategy for preparing silver nanocapsules with pNIPAM-*co*-AAc hydrogel cores.

**Synthetic strategy.** Scheme 1 depicts the strategy to prepare the silver nanocapsules with pNIPAM-*co*-AAc hydrogel cores. The steps include (i) synthesis of the pNIPAM-*co*-AAc hydrogel core, (ii) growth of THPC gold seeds around the hydrogel core, and (iii) growth of the silver nanocapsule around the hydrogel core by the reduction of silver nitrate onto the gold seeds, which act as templates. Note that the concentration of the sodium citrate during the galvanic replacement step determines whether the synthesized silver nanocapsule is porous or complete. By employing a combination of seeded-growth methods and SFEP techniques, we were able to facilitate the reliable growth of silver nanocapsules using pNIPAM-*co*-AAc particles as core templates [74–75]. By judiciously varying the reaction conditions, we were able to tune the silver shell thickness, the structural morphology, and the optical absorption of the silver nanocapsules, as discussed in the following sections.

**Figure 1:**
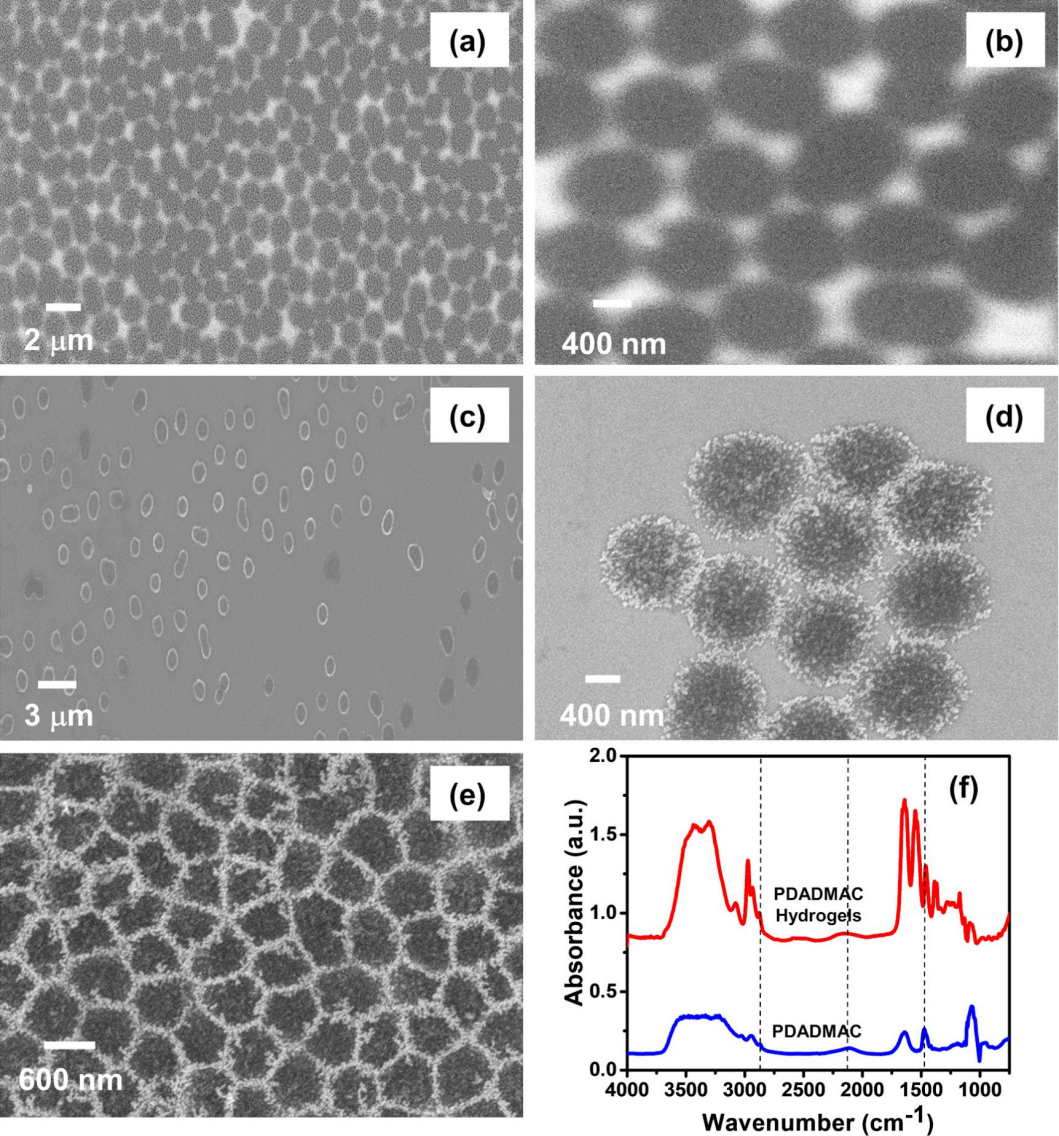
SEM images of (a) bare pNIPAM-*co*-AAc hydrogel particles (low magnification), (b) bare pNIPAM-*co*-AAc hydrogel particles (high magnification, ≈800 nm in diameter), (c) THPC gold seeds on hydrogel particles (low magnification), (d) THPC gold seeds on hydrogel particles (high magnification, ≈850 nm in diameter), (e) close packing of THPC gold-seeded hydrogel particles; and (f) Fourier transform infrared (FTIR) spectra of pure pDADMAC and pDADMAC-modified p(NIPAm-*co*-AAc) hydrogel particles.

**Size and morphology of the pNIPAM-*co*-AAc hydrogel cores.** SEM measurements were taken to determine the size of the hydrogel cores as well as to gain insight into the morphology of the particles. Figure 1a shows representative SEM images of the pNIPAM-*co*-AAc hydrogel core particles synthesized in this study. The hydrogel core particles exhibited polydisperse character; the lack of rigidity in the structure of pNIPAM-*co*-AAc proved difficult to illustrate monodispersed colloidal hydrogels. Figure 1b shows an image of the pNIPAM-*co*-AAc hydrogels at high magnification. Using statistical image analysis of the SEM images, the typical size of the hydrogels was determined to be 787 ± 81 nm.

To bind the negatively charged THPC gold nanoparticles to the surface of the hydrogel particles [78], we needed the hydrogel particle surfaces to be positively charged. For this modification, we chose poly(diallyldimethylammonium chloride) *M*_W_: 100,000 (pDADMAC), which has been previously used to attach noble metal seeds to hydrogel cores [79–81]. To verify the successful functionalization of pDADMAC on the hydrogel particle surface, we analyzed the particles with Fourier transform infrared (FTIR) spectroscopy. Figure 1f shows the absorption spectra of pure pDADMAC and the modified hydrogel particles. The existence of stretching associated with the CH_3_ components at ≈1465 cm^−1^ and ≈2900 cm^−1^ in addition to C–N stretching at ≈2100 cm^−1^, provides evidence of the presence of pDADMAC [82]. The images shown in Figure 1c,d illustrate the attachment of THPC gold seeds onto the pDADMAC-modified surface of the hydrogel core particles. Upon seeding, the diameters of the particles appear to increase in size to 866 ± 97 nm, as judged by the SEM images in Figure 1. Notably, the slight distortions in the images of the bare hydrogel composites (i.e., blurring and stretching apparent in Figure 1a and 1b) arise from surface charge build up from the SEM beam. Conversely, the conductive surfaces of the composites bearing gold seeds and silver shell particles undergo no charge build up, and consequently give clear, nondistorted images (see Figure 1c–e). Interestingly, the THPC gold-seeded pNIPAM-*co*-AAc hydrogel core particles assemble into a close-packed arrangement (Figure 1e), with no consistently uniform shape due to the soft hydrogel core, as discussed previously. As expected, without the addition of pDADMAC onto the surface of the hydrogel particles, insufficient seeding of the hydrogel cores was observed (data not shown). Thus, the FTIR spectra demonstrate the pDADMAC modification of the hydrogel core surface, and the SEM images demonstrate the attachment of colloidal THPC gold onto the pDADMAC-modified hydrogel core particles.

**Figure 2:**
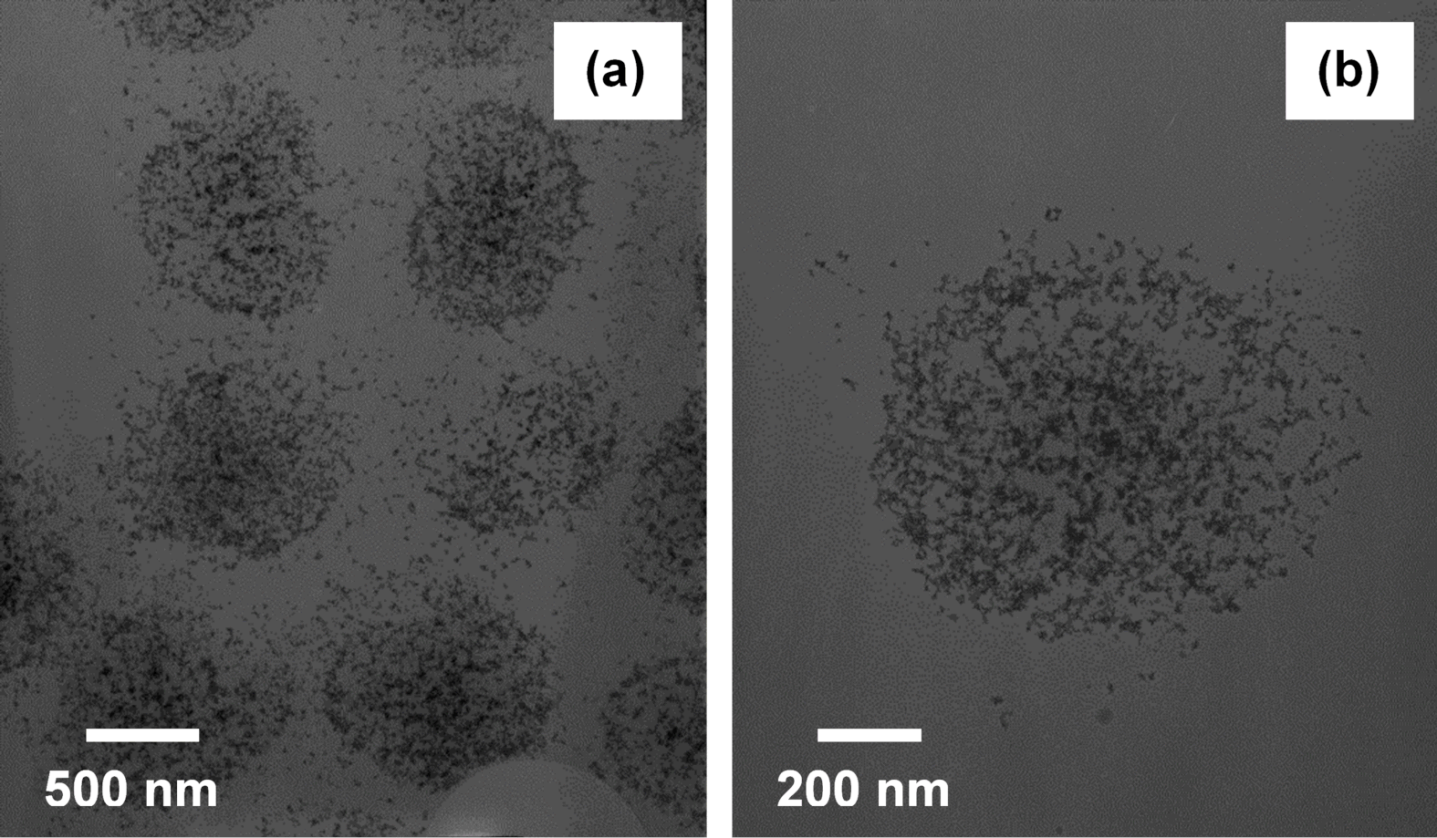
TEM images of (a) THPC gold seeds on pDADMAC-modfied hydrogel particles (low magnification) and (b) THPC gold seeds on pDADMAC-modified hydrogel particles (high magnification, ≈850 nm in diameter).

To analyze the structure and morphology of the THPC gold-seeded pNIPAM-*co*-AAc hydrogel core particles in greater detail, we employed transmission electron microscopy (TEM). Figure 2a shows representative images of the THPC gold-seeded on pDADMAC-modified pNIPAM-*co*-AAc hydrogel core particles. The TEM measurements reveal high populations of THPC gold seeds uniformly distributed over the modified hydrogel surfaces. The images reveal the manner in which the particles are attached, consisting of individually separated particles on the surface. In addition to the hydrogel cores themselves being polydisperse, uneven drying of the particles under high-vacuum conditions in the TEM might further increase the polydispersity of the sample. Figure 2b shows a high-magnification image of a THPC gold-seeded pDADMAC-modified pNIPAM-*co*-AAc hydrogel core particle. Due to insufficient electron density in the pNIPAM-*co*-AAc, the polymer core is not visible by TEM. However, due to the consistent size measurements of the gold clusters, and the absence of randomness in the gold nanoparticle distribution, we can conclude that there is a soft hydrogel polymer core onto which the gold seeds are attached. Furthermore, these results are consistent with SEM measurements.

**Thermo-responsive behavior of the pNIPAM-*co*-AAc hydrogel cores.** The swelling and collapsing behavior of hydrogels is well-documented and has been shown to occur due to changes in the hydrogen bonding between water and hydrophilic sites along the hydrogel polymer backbone (e.g., –C=O and –NH) [58,63,83]. The loss of hydrogen bonds between water molecules and the amide groups of a pNIPAM hydrogel above its LCST leads to the collapse of the hydrogel polymer due to increased hydrophobic interactions in the polymer hydrogel network [58]. Ionizable groups such as AAc and AAm are grafted into the polymer backbone to increase electrostatic interactions. The introduction of AAc, which has ionizable COOH groups, induces electrostatic hydrogen-bonding interactions and consequently higher osmotic swelling pressure, thereby increasing the particle size to a more swollen state below the LCST [84]. Due to these same induced interactions, the LCST of highly ionized microgels is also shifted to higher temperatures compared to particles without the co-monomer. This shift in LCST can be controlled by adjusting the co-monomer concentration [85]. The LCST of pure NIPAM hydrogels is constant at ≈30 °C; therefore, the ability to adjust the LCST to biologically relevant temperatures of ≈34–45 °C is made possible by chemical modification of the polymer backbone [83,85].

**Figure 3:**
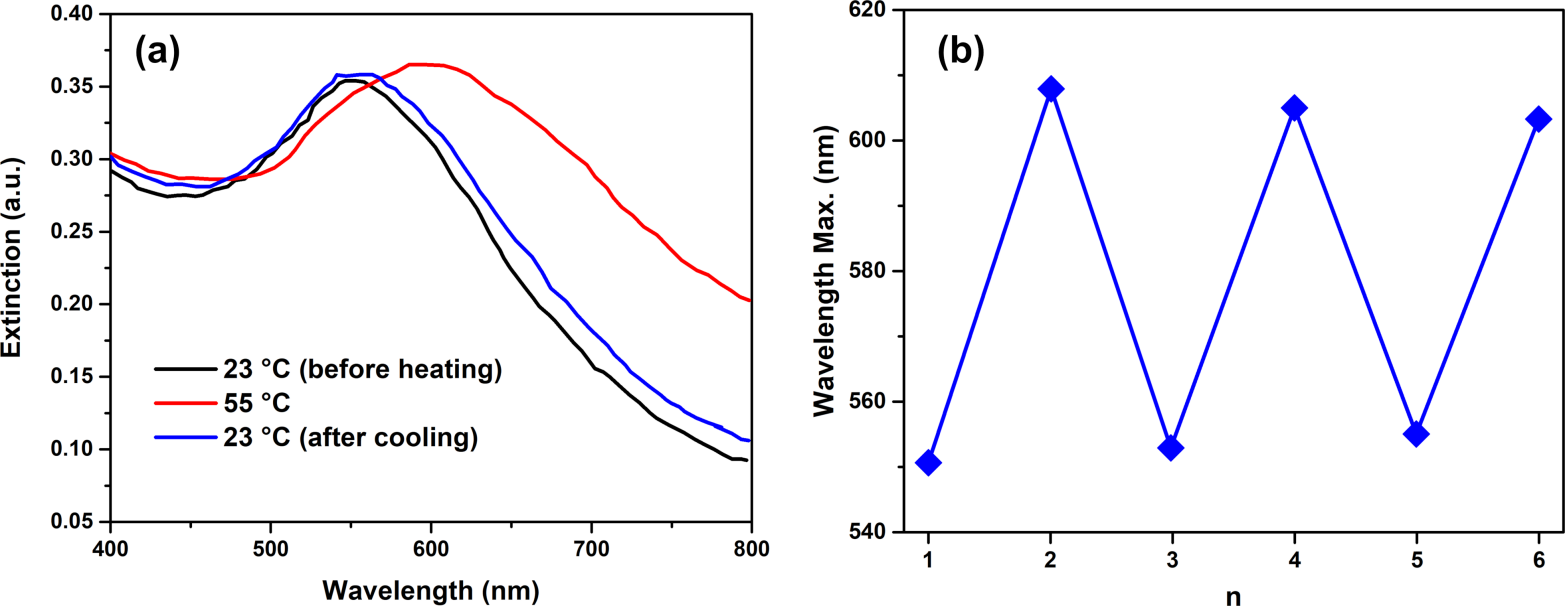
(a) UV–vis spectra of THPC gold seeds on swelled and collapsed pNIPAM-*co*-AAc hydrogel particles at 23 °C and 55 °C, respectively; (b) Swelling and collapsing of THPC gold seeds on pNIPAM-*co*-AAc hydrogel particles at 23 °C and 55 °C as a function of extinction maximum.

To demonstrate the thermo-responsive behavior of the soft hydrogel polymer core particles, we performed UV–vis spectral analysis on the THPC gold-seeded hydrogel core particles at selected temperatures. Figure 3 shows the UV–vis spectra of the THPC gold-seeded hydrogel core particles at two biologically relevant temperatures. From the extinction spectra, the behavior of the hydrogel core particles was observed from room temperature to 55 °C. At room temperature, the particles were in their swollen state (i.e., their largest size). As the temperature was increased, water was expelled from the particle, causing the core to collapse and leading to a smaller size. Thermochromic effects in the particles can be detected by examining the extinction maximum shift from 550 to ≈600 nm, corresponding to the plasmon band of the THPC gold seeds. The red-shift observed in the extinction spectra provides evidence of the gold particles coalescing on the surface of the hydrogel core as the temperature increases, an effect consistent with previous studies [46,86]. Cooling and heating cycles were repeated several times to demonstrate the swelling and collapsing behavior of the hydrogel core particles. Figure 3b illustrates the maximum extinction wavelength of the THPC gold-decorated pNIPAM-*co*-AAc nanoparticles as a function of the cooling and heating cycles. The particles were able to undergo up to six heating and cooling cycles before showing signs of structural degradation, as indicated by visible aggregation and loss of the extinction peak intensity. At elevated temperatures, the hydrogel cores are in a desolvated state as indicated by the swollen and collapsed architectures shown in Figure 3. Furthermore, a reduction in the overall surface charge distribution is expected to accompany the increase in temperature, which allows the hydrogel to collapse inside the capsule. Importantly, the data confirm the thermo-responsive nature of the soft hydrogel cores used in the study at biologically relevant temperatures.

**Figure 4:**
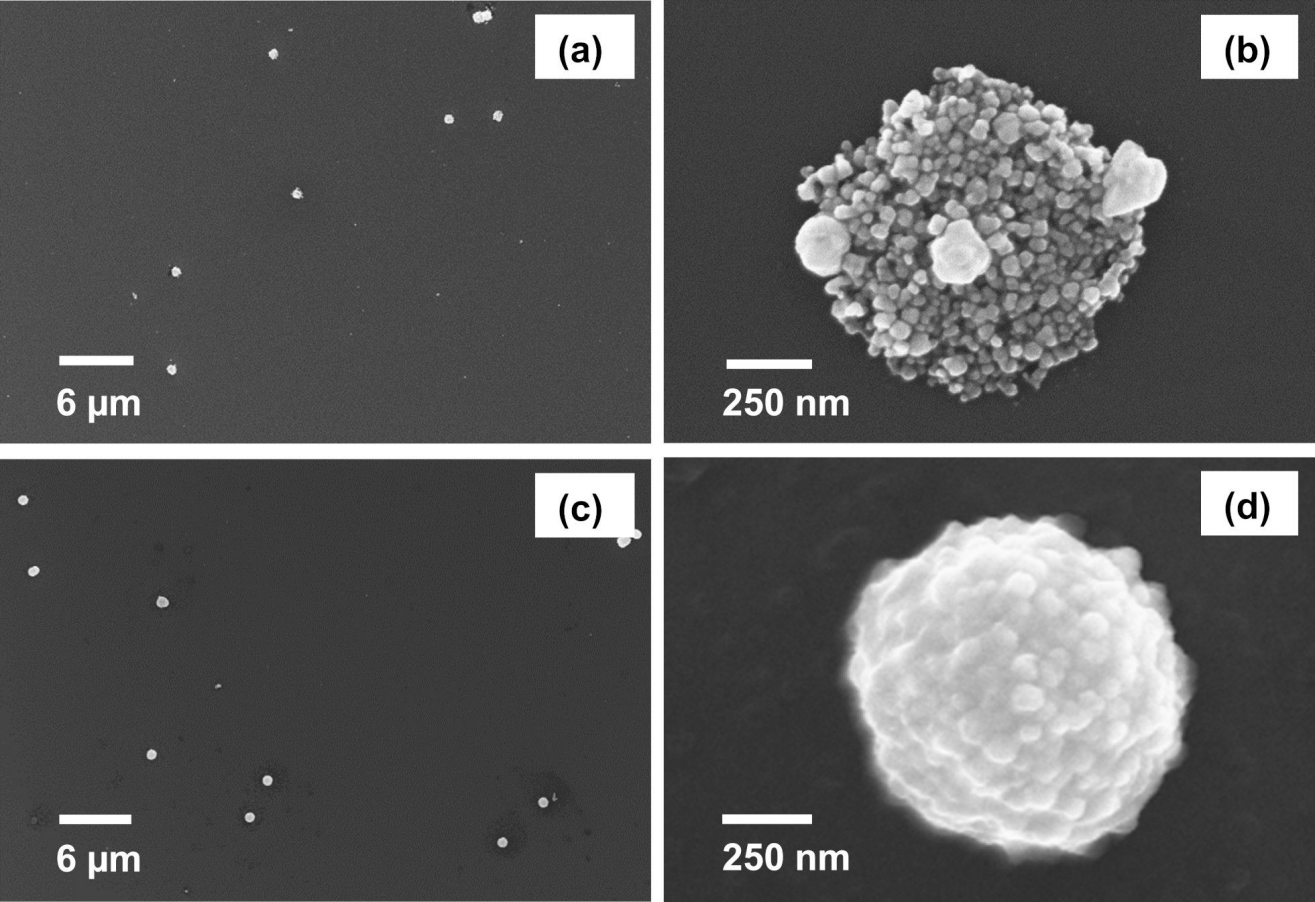
SEM images of (a, b) porous silver nanocapsules with hydrogel cores and (c, d) complete silver nanocapsules with hydrogel cores.

**Size and morphology of the porous silver nanocapsules with pNIPAM-*co*-AAc hydrogel cores.** We employed SEM to confirm the existence of the silver nanocapsules surrounding the hydrogel core templates and examine the size and morphology of the particles. Figure 4 shows images of porous silver nanocapsules with pNIPAM-*co*-AAc hydrogel cores and continuous silver nanocapsules with pNIPAM-*co*-AAc hydrogel cores. The Au nanoparticles act as nucleation sites (templating agents) for the growth of the Ag shells. Without these templating agents, the core–shell particles fail to form, leading to the exclusive formation of free metal particles and aggregated particles. Additionally, THPC can act as both a reducing agent and a stabilizing ligand [77]. THPC is known to produce small, uniformly spherical Au nanoparticles, which is critical for the growth of morphologically smooth Au nanoshells [87]. Given the successful use of THPC-Au seeds for growing Au shells and the similarities between gold and silver, we anticipated that THPC-Au seeds would also serve as reliable templating agents for the growth of Ag nanocapsules [77].

The growth of a continuous silver metal nanocapsule on the THPC gold hydrogel core was accomplished using an appropriate reducing agent, such as formaldehyde, following a seeded-growth method [77]. Figure 4a and Figure 4c confirm that the particles are well-dispersed after nanocapsule growth with no aggregation, and the diameters of 929 ± 42 nm and 920 ± 58 nm were determined for the porous and smooth Ag nanocapsules, respectively. However, the surface morphology of the porous nanoshells is rough (see Figure 4b). Possible reasons for the roughness include destabilizing intercalation of the salt precursor into the polymer matrix and insufficient nucleation sites, leading to lengthy time periods needed for nanocapsule growth. To circumvent these issues and accomplish smooth, continuous nanocapsule growth, we utilized sodium citrate to assist in the stabilization of the core template along with silver nitrate to increase the rate of nanocapsule growth. This approach proved to be successful in growing complete silver nanocapsules, as demonstrated in Figure 4d. The SEM images of the complete silver nanocapsules (Figure 4d), with their smoother surface, contrasts that of the porous silver nanoshells (Figure 4b). Thus, simply increasing the sodium citrate concentration alters the particle structure from a porous, irregular architecture to a complete, markedly smoother nanocapsule.

In this study, we adopted a seed-mediated approach for templating the hydrogel particles and subsequently growing the silver shells. This method takes advantage of the charge distribution dominant on the particle surface between the polymer layer and the hydrogel, as well as the negatively charged THPC-Au seed particles and the positively charged pDADMAC layer [88]. As such, the stable attachment of the Ag particles to the polymer in aqueous solution was demonstrated by repeated cycles of centrifuging and redispersing the particles via sonication in water. Notably, the particles with continuous Ag shells exhibited greater stability than the particles with porous Ag shells. Additionally, the samples having porous Ag shells were often accompanied by free silver particles in solution when redispersed in water (see Figure 4a and Figure 4b). In contrast, Figure 4c and Figure 4d illustrate the absence of smaller, free silver particles after aqueous redispersion of the samples having continuous Ag shells.

**Figure 5:**
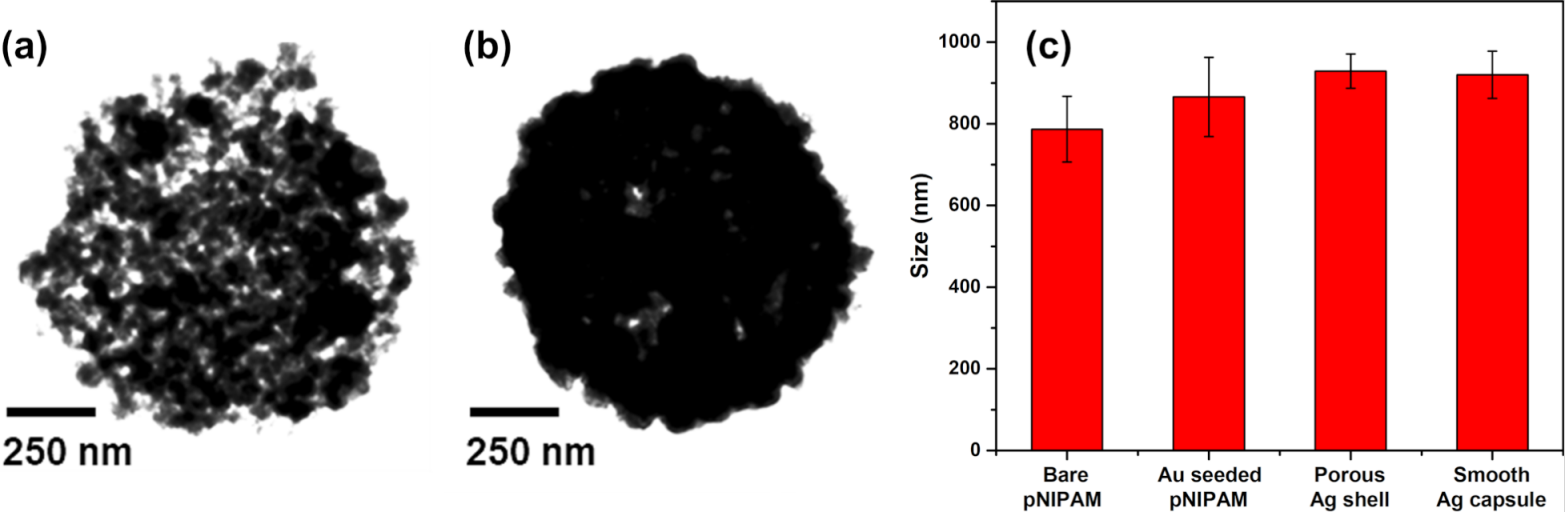
TEM images of (a) porous silver nanocapsules with hydrogel cores (≈930 nm in diameter), (b) silver nanocapsules with hydrogel cores (≈920 nm in diameter), and (c) stepwise particle size (diameter) distribution plot.

In addition to SEM studies, TEM analysis was also performed to further demonstrate the successful growth of continuous silver nanocapsules. Figure 5a shows porous silver nanocapsules with total diameters of ≈930 nm, which correspond to a shell thicknesses of ≈40 nm. Porous structures were obtained when sodium citrate concentrations were low. By increasing the concentration of sodium citrate in the reaction, smooth continuous silver nanocapsules were formed (Figure 5b) with overall diameters of ≈920 nm and capsule thicknesses of ≈40 nm. Note also the stepwise size progression of the particles shown in Figure 5c.

**Figure 6:**
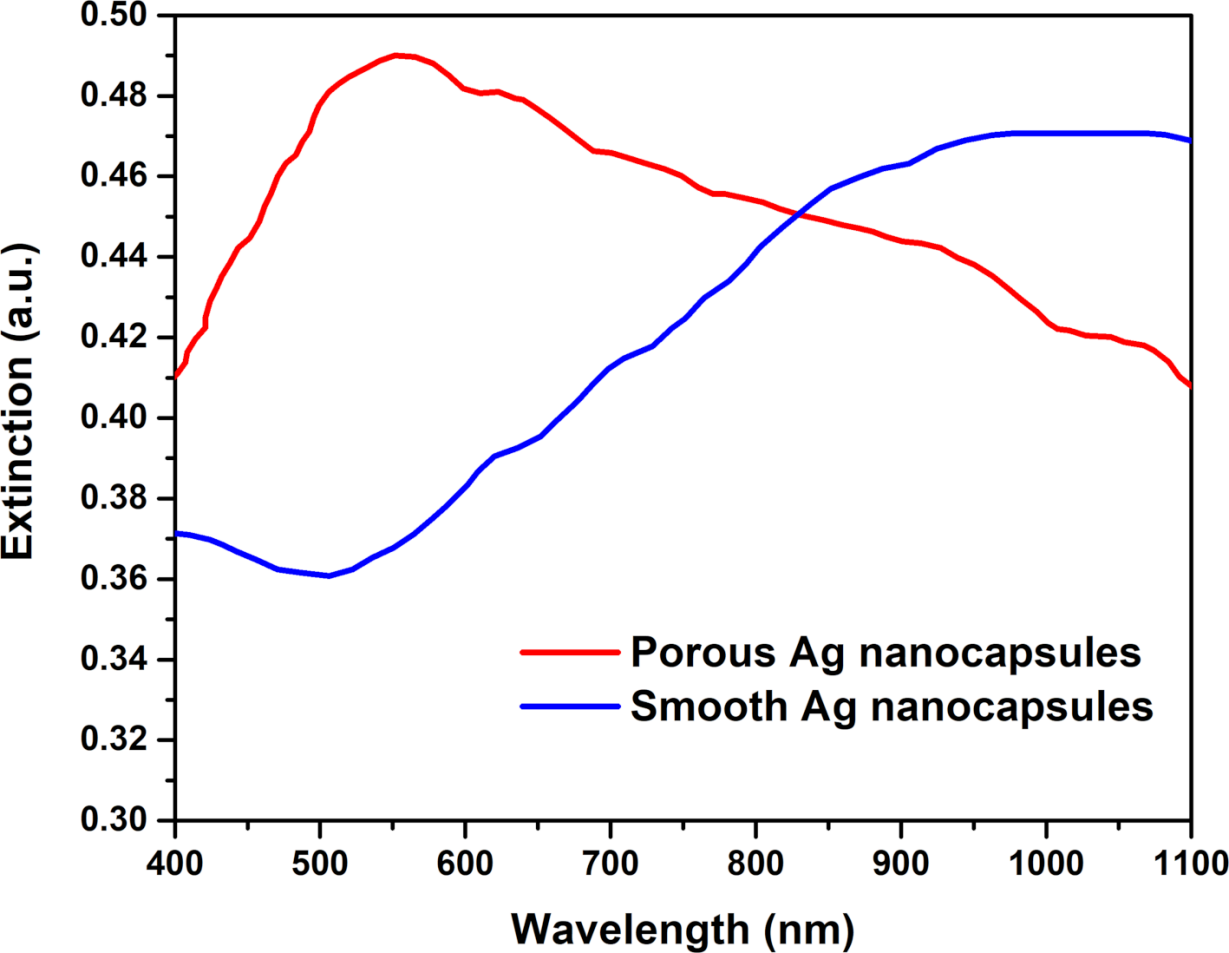
UV–vis spectra of porous silver nanocapsules with hydrogel cores (≈930 nm in diameter) and complete silver nanocapsules with hydrogel cores (≈920 nm in diameter).

**Optical properties of the porous silver nanocapsules with pNIPAM-*co*-AAc hydrogel cores.** Figure 6 shows the UV–vis extinction spectra of the silver nanocapsules. The behavior (i.e., positions, intensities, and broadening) of the absorption bands can be modeled using Mie theory [89]. The porous silver nanocapsules produced a maximum extinction band at ≈550 nm. The shape of the band for the porous shell is significantly different from that of the continuous, smooth silver nanocapsules. The primary difference exists in the region at ≈500 nm, which suggest the presence of silver nanoscale spherical aggregates rather than complete shells in the porous silver nanocapsules [90–91]. This result is consistent with the SEM and TEM images, which indicate the existence of discontinuous nanocapsules; that is, there are multiple silver nanoparticles surrounding the hydrogel cores in these samples.

The appearance of the extinction maximum for the complete silver nanocapsules at ≈950–1050 nm is consistent with that of a complete nanoshell [51,92–93]. The broadening of the spectra is likely the result of several contributing factors, such as polydispersity of the hydrogel core particles, capsule roughness, variable capsule thicknesses, and/or overlap of multipole surface plasmon resonances. The contribution from overlapping multipole surface plasmon resonances is perhaps the most dominant factor due to the large size of the nanocapsules. Thus, the UV–vis measurements along with the SEM and TEM images definitively illustrate the successful preparation of silver nanocapsules with both porous and complete silver shells.

## Conclusions

By utilizing a seeded-growth method and surfactant-free emulsion polymerization, we demonstrated a reliable synthesis of silver nanocapsules encapsulating thermo-responsive pNIPAM-*co*-AAc hydrogel cores. The 800 nm silver nanocapsules with a capsule thickness of ≈50 nm were characterized by SEM, TEM, and UV–vis spectroscopy. The optical properties of the THPC gold-seeded hydrogel core particles were dependent upon temperature and yielded thermochromic responses in the visible spectral regions (≈550 to ≈600 nm) when the temperature was varied from 23 to 55 °C. In contrast, the extinction spectra of the nanocapsules varied with porosity. The porous silver nanocapsules exhibited extinction maxima in the visible spectral regions (λ_max_ ≈ 550 nm with tailing into the NIR), while complete nanocapsules exhibited extinction maxima in the NIR spectral regions (λ_max_ ≈ 950–1050 nm). The methods described in this study, detailing the preparation of silver nanocapsules using pNIPAM-*co*-AAc hydrogel cores, provides a platform for potential “smart delivery” vehicles capable of responding to external stimuli (e.g., light, pH, and temperature). The delivery vehicles based on gold-coated hydrogels, having both porous shells and tissue-transparent NIR extinctions, are the objects of future research.

## Experimental

**Materials.** The following chemicals were purchased from the indicated suppliers and used without further modification: formaldehyde, sodium hydroxide, potassium persulfate (KPS, 99%), ammonium hydroxide (30% NH_3_), nitric acid, hydrochloric acid (all from EM Science), potassium carbonate (from J. T. Baker), poly(diallyldimethylammonium chloride) *M*_W_: 100,000 (pDADMAC), tetraethylorthosilicate (TEOS), tetrakis(hydroxymethyl)phosphonium chloride (THPC), 3-aminopropyltrimethoxysilane (APTMS, all from Aldrich), silver nitrate (AgNO_3_, Strem), and ethanol (Aaper). *N*-Isopropylacrylamide (NIPAM, 99%) and acrylic acid (AAc, 99.5%) were obtained from Acros. Water was purified to a resistivity of 18.2 MΩ cm (Academic Milli-Q Water System; Millipore Corporation) and filtered using a 0.22 μm filter to remove any impurities. All glassware and equipment used were first cleaned using aqua regia (3:1, HCl/HNO_3_), then cleaned in a base bath (saturated KOH in isopropyl alcohol) and lastly, rinsed with Milli-Q water prior to use.

**Preparation of pNIPAM-*co*-AAc hydrogel particles.** Colloidal pNIPAM-*co*-AAc hydrogel particles were prepared using the SFEP method [74–76]. Briefly, a two-necked round-bottomed flask (RBF) equipped with a reflux condenser and an inlet for argon gas was filled with 50 mL of water. NIPAM (0.225 g; 1.98 × 10^−3^ mol) and the cross-linker BIS (0.025 g; 1.62 × 10^−4^ mol), were added to the RBF containing water and stirred for 30 min. Due to the possibility of oxygen intercepting radicals and disrupting the polymerization process, argon gas was bubbled through the stirred solution for 30 min to remove any oxygen. Blanketed with argon, the mixture was heated to 70 °C in an oil bath and then KPS (0.111 g; 4.11 × 10^−4^ mol) was added to initiate the polymerization. After 15 min, an aliquot of acrylic acid AAc (0.024 g; 3.33 × 10^−4^ mol) in 1.5 mL of water was added. Following vigorous stirring of the mixture for 4 h, the reaction mixture was subsequently cooled to room temperature and filtered through filter paper to remove any micrometer-sized impurities and/or any aggregated particles. To remove any unreacted materials and soluble side products, the filtered solution was centrifuged at 25 °C for 1 h at 3500 rpm two times, discarding the supernatant each time. The purified hydrogel particles were then suspended in 40 mL of water and kept at room temperature. The hydrogel particles showed no signs of visible aggregation, and their size (≈800 nm in diameter) was controlled by adjusting the amount of initiator and the reaction time.

**Attachment of THPC gold seeds to pNIPAM-*co*-AAc hydrogel particles.** For the attachment of colloidal gold seeds onto the hydrogel particles, modification of the hydrogel surface was necessary. To accomplish this, pDADMAC (1 mg/mL) was added to a diluted solution of hydrogel particles. The solution was briefly sonicated for 5 min and allowed to further sit for 1 h at room temperature. The particles were then centrifuged at 3500 rpm for 1 h and redispersed in 20 mL of water. For complete purification of the particles, this cycle was repeated three times to remove any unreacted pDADMAC.

To facilitate the attachment of gold seeds, a THPC gold solution was prepared using a modified Duff method [77,87]. Typically, the procedure yielded Au seeds ≈2–4 nm in diameter, and the size could be varied by changing the initial gold salt concentration. It was observed that storing the colloidal solution in the refrigerator for three days helped obtain effective gold seed attachment. The THPC gold seeds were attached onto the hydrogel core particles using methods previously described [77–78]. Briefly, the THPC gold seeds were deposited onto the hydrogel core particles by adding 5 mL of a concentrated THPC gold solution to 0.25 mL of the surface modified hydrogel particles, and the mixture was allowed to sit overnight. The resultant Au-seeded hydrogel particles were centrifuged and redispersed in clean water to remove any unattached THPC gold seeds.

**Silver nanocapsule formation.** Following the work in [77], a silver growth solution was prepared by dissolving silver nitrate (0.003 g, 0.018 mmol) in 50 mL of water, and a solution of sodium citrate (0.002 g, 0.008 mmol) was prepared in 50 mL of water. To encapsulate the Au-seeded hydrogel particles with silver, 4 mL of silver nitrate solution and 1 mL of sodium citrate solution were stirred for 3 min, following which 0.25 mL of THPC gold-seeded hydrogel particles were added to the growth solution and stirred for another 3 min. Ammonium hydroxide (50 µL) and formaldehyde (25 µL) were simultaneously added to initiate reduction of silver nitrate to silver. Stirring was continued for 4 min to produce a complete silver nanocapsule with a broad extinction maximum at ≈950–1050 nm. Additionally, porous nanocapsules were formed by simply modifying the amount of sodium citrate solution used. To produce porous nanocapsules, the sodium citrate solution was changed from 0.008 to 0.004 mmol and added to the reaction.

**Characterization methods.** The overall morphology of the particles was analyzed using a LEO SEM instrument operating at an accelerating voltage of 15 kV and a JEM-2000 FX TEM (JEOL) operating at an accelerating voltage of 200 kV. Bare hydrogel particles, THPC gold-seeded hydrogel particles, and silver nanocapsules were deposited on clean silicon wafers and thoroughly dried at room temperature overnight before obtaining the SEM images. The images at low magnification were taken to demonstrate good dispersion. The image analysis for size distribution was carried out using ImageJ software. Bare pNIPAM-*co*-AAc hydrogel particles, THPC gold-seeded hydrogel particles, and silver nanocapsules were deposited on 300 mesh holey-carbon-coated copper grids and dried overnight before analysis by TEM. We utilized a Cary 50 Scan UV–vis optical spectrometer (Varian) equipped with Cary Win UV software over the wavelength range of 300–1100 nm to analyze the optical properties of the THPC gold-seeded hydrogels and silver nanocapsules. To examine the swelling and collapsing behavior of the THPC gold-seeded hydrogels, the solution of particles was heated in a cuvette over a temperature range of 23–55 °C. Once the particle temperature was equilibrated to the desired temperature, optical measurements of the particles were acquired. FTIR spectral data was obtained using a Nicolet Nexus FTIR 670 spectrometer. Aqueous solutions of the hydrogel particles and pDADMAC were directly deposited onto a silicon wafer and dried under vacuum. Spectra were acquired by scanning each sample 32 times with a background correction at a spectral resolution of 4 cm^−1^.
